# A Novel Role of IL13Rα2 in the Pathogenesis of Proliferative Vitreoretinopathy

**DOI:** 10.3389/fmed.2022.831436

**Published:** 2022-06-13

**Authors:** Hui Qi, Lijun Dong, Dong Fang, Lu Chen, Yun Wang, Ning Fan, Xingxing Mao, Wenyi Wu, Xiaohe Yan, Guoming Zhang, Shaochong Zhang, Hetian Lei

**Affiliations:** ^1^Shenzhen Eye Hospital, Shenzhen Eye Institute, Jinan University, Shenzhen, China; ^2^Department of Ophthalmology, Hunan Key Laboratory, National Clinical Research Center for Geriatric Disorders, Xiangya Hospital, Central South University, Changsha, China

**Keywords:** vitreous, retinal pigment epithelial cells, RNA sequencing, IL13Rα2, proliferative vitreoretinopathy

## Abstract

Proliferative vitreoretinopathy (PVR), an inflammatory and fibrotic blinding disease, is still a therapeutic challenge. Retinal pigment epithelial (RPE) cells dislodged in the vitreous play a central role in the PVR pathogenesis. To identify potential novel contributors to the pathogenesis of PVR, we investigated a profile of vitreous-induced changes in ARPE-19 cells by RNA sequencing. Bioinformatics analysis of the sequencing data showed that there were 258 genes up-regulated and 835 genes down-regulated in the ARPE-19 cells treated with human vitreous. Among these genes, there were three genes related to eye disease with more than threefold changes. In particular, quantitative PCR and western blot results showed that interleukin 13 receptor (IL13R)α2 that is over-expressed in a variety of cancers was up-regulated more than three times in the vitreous-treated ARPE-19 cells. Immunofluorescence analysis indicated that interleukin-13 receptor subunit α2 (IL13Rα2) was highly expressed in ARPE-19 cells within epiretinal membranes from patients with PVR. Importantly, blocking IL13Rα2 with its neutralizing antibody significantly inhibited vitreous-induced contraction of ARPE-19 cells, suggesting a novel role of IL13Rα2 in the PVR pathogenesis. These findings will improve our understanding of the molecular mechanisms by which PVR develops and provides potential targets for PVR therapeutics.

## Introduction

Proliferative vitreoretinopathy (PVR) refers to the retinal re-detachment caused by extensive contraction and traction of the proliferative membrane on the surface of the retina and behind the vitreous after reattachment of rhegmatogenous retinal detachment (RRD) (1–4). It is the most common cause of failure to repair RRD (2). The growth and contraction of cell membranes in the vitreous cavity and on both sides of the surface of the retina, and intraretinal fibrosis are characteristics of PVR. PVR occurs in 8–10% of patients undergoing primary retinal detachment correction surgery, and in 40–60% of patients with open global injury (5, 6). At present, repeated surgery is the only option for patients with PVR (3, 7). But recurrent detachment and retinal damage caused by the PVR process itself lead to poor visual effects of the surgery. In the past period of continuous development of vitreous surgery technology, the incidence of PVR in prospective studies has remained unchanged (8, 9).

Fibrotic epiretinal membranes (ERMs) are composed of extracellular matrix such as collagen and fibronectin and cells such as retinal pigment epithelial (RPE) cells, fibroblasts, glial cells, and macrophages (3, 10–12). Almost all risk factors for PVR are related to the diffusion of RPE cells in the vitreous or the destruction of the blood–eye barrier (6). In the process of retinal tear in PVR patients, the detached RPE cells will contact the vitreous, causing the vitreous to stimulate the migration of RPE cells on the surface of the retina. At the same time, the inflammatory mediators and blood released by the retinal tear promote the production of collagen by RPE cells, which further lead to the formation and contraction of ERM (13). Due to the role of RPE cells and glial cells in the vitreous membrane in the pathogenesis of PVR, RPE cells have been widely used in the study of the pathogenesis of PVR (14, 15).

The purpose of this study was to investigate the differentially expressed genes (DEGs) of human RPE cells induced by vitreous and thereby to identify potential therapeutic targets.

## Materials and Methods

### Major Reagents

Primary antibodies against β-actin (Cat. 4970 1:2,000), pan Keratin (Cat. 4545S, 1:200), and interleukin13 receptor subunit α2 (IL13Rα2) (Cat. 85677S 1:1,000) were bought from the Cell Signaling Technology (Danvers, MA, United States), a neutralizing antibody for IL13Rα2 was purchased from the R&D Systems (Cat. AF146), and a primary antibody against KI67 (Cat. ab243878, 1:200) was purchased from the Abcam (Danvers, MA, United States). Horseradish peroxidase-conjugated goat anti-rabbit IgG (Cat. SA00001-2, 1:5,000) was ordered from the Proteintech (Danvers, MA, United States). Fluorescent labeled secondary antibodies of rabbit (Cat. A21206) or mouse (Cat. WA316324) were bought from the Thermo Fisher Scientific (Waltham, MA, United States).

### Patient Vitreous and ARPE-19 Cells Culture

Before launching the project, the ethics approval of the clinical research ethics committee of Jinan University was obtained. A written informed consent was signed by each patient before vitreous samples were harvested from patients with or without PVR (HV).

ARPE-19 purchased from American Type Culture Collection (Manassas, VA, United States), and was cultured in the DMEM/F12 medium with 10% fetal bovine serum (FBS) and 1% penicillin G sodium (100 units/ml) and streptomycin (100 mg/ml) in a humidified incubator at 37°C with 5% CO_2_ (16).

### Porcine Vitreous

Porcine vitreous was taken from fresh porcine eyes, frozen at a –80°C freezer. The eyeballs were dissected on ice and the isolated vitreous was diluted at 1:3 in DMEM/F12 and filtered for sterilization (17).

### Cell Proliferation Assay

Cells were cultured a 6-well plate with slides in the DMEM/F12 supplemented with 10% FBS. After the cells were completely attached to the slide, the medium was changed to DMEM/F12 only. Then the cells were treated with vitreous with Ki67 for a proliferation assay. The Ki67 primary antibody was incubated at 4°C overnight, and the fluorescent secondary antibody was incubated for 1 h in the dark at room temperature. Finally, the cells were stained with 4’,6-diamidino-2-phenylindole (DAPI) for 10 min, and the slides were mounted for photographs in a NIKON Ti2-E fluorescence microscope (18, 19).

### Cell Migration Assay

Cells were grown to confluence in a 24-well plate before the cell monolayer was scratched with a 200-μl-pipet tip to create a wound. After washing the cells twice with phosphate-buffered saline (PBS), either DMEM/F12, or vitreous (1:3 dilution in DMEM/F12) was added. Photographs were taken to record the width of the wounds at the beginning of the experiment, and then they were taken again 24 h later. After at least three independent experiments were conducted, Adobe Photoshop CC 2018 software was used to analyze the wound healing areas (20, 21).

### Contraction Assay

Cells were trypsinized, counted, and centrifuged at 800 rpm for 5 min. The cell pellets were re-suspended in a collagen I solution (Cat. A10483, Gibco, United States) (pH 7.2) on ice, and the collected cells were diluted to 1 × 10^6^ cells/ml in the collagen solution, which were transferred to the wells (300 μl/well) in a 24-well plate that had been incubated with 5 mg/ml bovine serum albumin (BSA)/PBS overnight. The plates were moved into a 37°C incubator for 90 min to allow the collagen to solidify, where upon the collagen gel was overlaid with 0.5 ml of either DMEM/F12, vitreous (1:3 dilution in DMEM/F12), or vitreous plus a neutralizing antibody against IL13Rα2 (1 μg/well). After 48 h, the diameter of the gel was measured and calculated. The data of three independent experiments were used for statistical analysis (21–23). Neutralizing the IL13RA2 receptor using neutralizing antibodies in collagen gel contraction is the same as the contraction assay.

### RNA Sequencing

Serum starved ARPE-19 cells at 70–80% confluence were treated with human vitreous diluted at a ratio of 1:3 in the DMEM/F12 medium for 24 h. The treated cells were then harvested by Trizol for RNA isolation using an OMEGA kit (R6834). Subsequently, a Complementary DNA (cDNA) library was established and the quality and integrity of the RNA were examined by a NanoDrop analysis. RNA sequencing was performed by NovaSeq 6000. The differential genes were screened to satisfy | log2FC| ≥ 1 and *p* < 0.05, and genes were further screened to identify significantly differentially expressed genes related to eye diseases (24, 25).

Notably, Gene Ontology (GO) and Kyoto Encyclopedia of Genes and Genomes (KEGG) are two databases, and enrichment analysis is an integrated calculation of the functional information in the database. The full name of GO database analysis is Gene Ontology, and it divides the function of genes into three parts: cellular component (CC), molecular function (MF), and biological process (BP). Using the GO database, we can get the disease correlation of the target gene at the three levels of CC, MF, and BP. KEGG database analysis is a kind of pathway-related database to study the various pathways of the human body that genes participate in (24, 25).

### Quantitative PCR (qPCR) Analysis

Total RNA in the RPE cells treated by vitreous was extracted using an OMEGA kit (R6834), and then reverse transcribed to construct a cDNA library using a kit (RR036A, Takara). The differential expression of eye disease-related genes was analyzed by qPCR using a kit (RR820A, Takara). Primer sequences: IL13RA2 forward (F): 5’-GGGCATTGAAGCGAAGATACA-3’; IL13RA2 reverse (R): 5′-GCCCAGGAACTTTGAACTTCTG-3′ (26); APCDD1 forward (F): 5′-TCCTGCTCAGATACCTGTTCC-3′; APCDD1 reverse (R): 5′- GTGATGGCACTGTGACTCCT-3′; CD180 forward (F): 5′-AACCTAAGCCTGAACTTCAATGG-3′; CD180 reverse (R): 5′-GCCAGAGAGACTGAGTAGTAGAG-3′.

### Western Blot

When cells reached 70–80% confluence, they were serum-starved for 24 h, and then treated with or without vitreous from patients (diluted 1:3 in DMEM/F12) for 24 h. After two washes with ice-cold PBS, cells were lysed in a cell lysis buffer for 30 min. The cell lysates were then clarified by centrifugation at 13,000 rpm for 10 min at 4°C. The total proteins were quantitated using a BCA kit (Cat. KGP250, keyGen, China), and the samples were boiled for 5 min. Proteins were separated by 10% sodium dodecyl-sulfate-polyacrylamide gel electrophoresis (SDS-PAGE), transferred to polyvinylidene difluoride (PVDF) membranes, and then subjected to western blot analysis using desired antibodies. Signal intensity was determined by densitometry using the Image J Software (22).

### Immunofluorescence

This experiment was performed as described previously (27, 28). Briefly, the tissues on slides were fixed with 4% paraformaldehyde (PFA) for 10 min at room temperature, blocked with 3% FBS for 30 min at room temperature, and then incubated with primary antibodies (anti-IL13Rα2 antibody or non-immune IgG at 1:200 dilution) at 4°C overnight. After thorough washes, the secondary antibody at a ratio of 1:1,000 was incubated at room temperature for 1 h in the dark. Next, the DAPI staining at a ratio of 1:1,000 was performed in the dark for additional 10 min. Finally, after the slides were washed thoroughly, they were mounted for photograph in a NIKON Ti2-E fluorescence microscope (27, 28).

### Statistics

Data from three independent experiments were used for statistical analysis with ordinary one-way ANOVA. The *p* value less than 0.05 was considered to be a significant difference (29).

## Results

### RNA Sequencing Analysis Reveals That There Are 258 Genes Up-Regulated and 835 Genes Down-Regulated in Vitreous-Treated Retinal Pigment Epithelial Cells

Treatment of RPE cells with vitreous *in vitro* was to mimic PVR pathogenesis *in vivo*, and vitreous from human, bovine, and experimental rabbits is able to enhance cell proliferation, survival, and contraction (23, 30, 31). We then tested if vitreous from porcine could also have a similar bioactivity. Results showed that porcine vitreous was also capable of enhancing cell proliferation as shown in Supplementary Figures 1–3. These data are consistent with our previous findings that vitreous enhances PVR-related cellular events (13).

**FIGURE 1:**
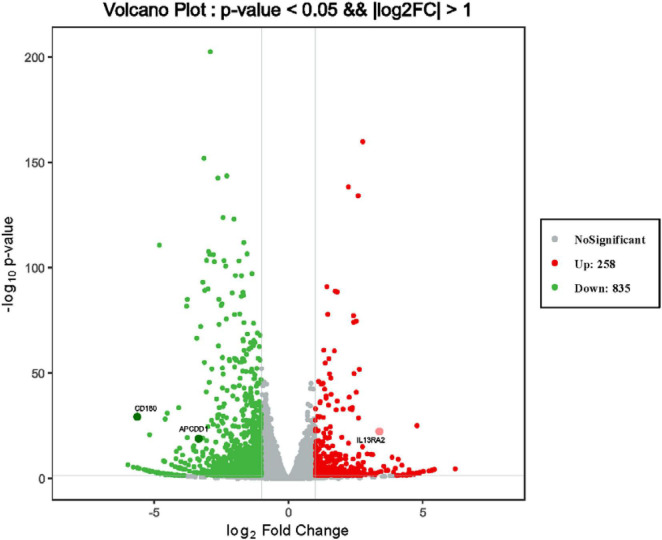
Differentially expressed genes (DEGs) of retinal pigment epithelial (RPE) cells induced by the control group and vitreous. Results of a volcano plot chart of the differentially expressed genes were identified from the control and vitreous-treated group.

**FIGURE 2:**
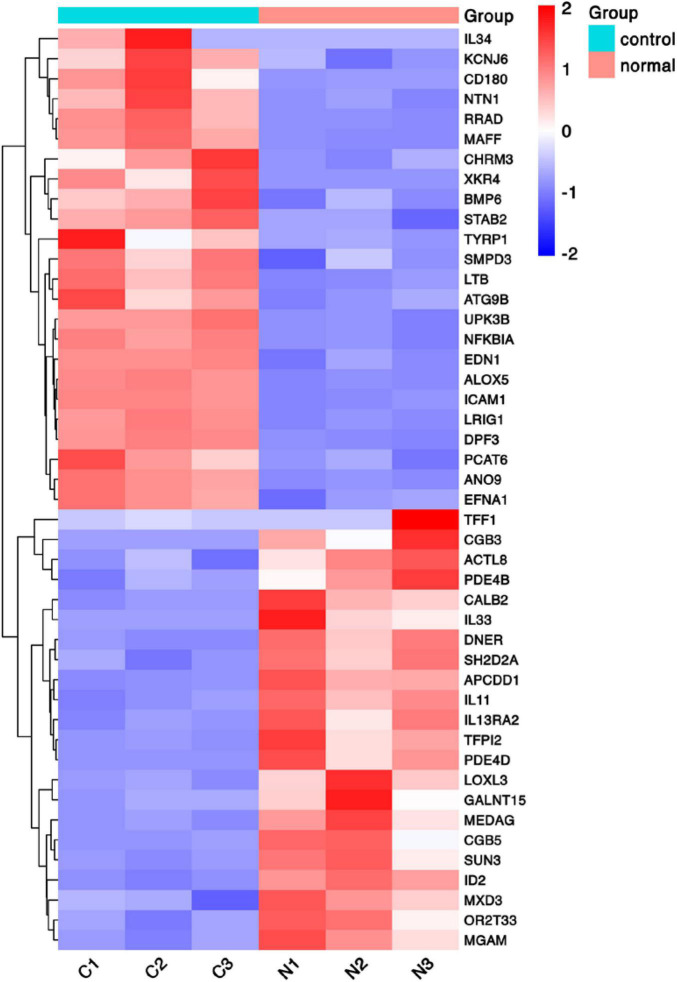
Heat map of hierarchical clustered genes. A heat map of hierarchical clustered genes. In total, there were 46 genes from the genomics data showing a significantly aberrant expression (at least two-fold change), *p* < 0.05. In the clustering analysis, red and blue regions indicate the up-regulated and down-regulated genes, respectively. C1, C2, C3, and N1, N2, N3 represent the three independent repetitions of the control group and the vitreous induction group, respectively.

**FIGURE 3:**
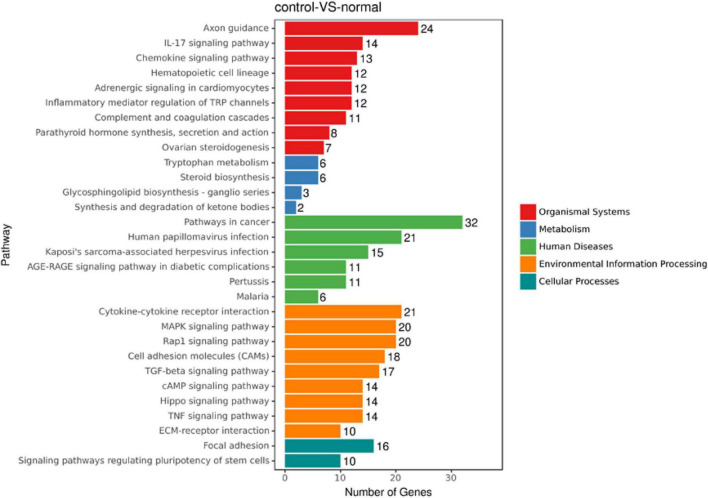
An enrich mean analysis of pathways for DEGs based on the KEGG database. An enrich mean analysis of pathways for DEGs based on the KEGG database. The horizontal and vertical axes represent the enrichment score of – log *p* and the pathway category, respectively.

To discover novel genes contributing to PVR pathogenesis, we next treated ARPE-19 cells with human vitreous for transcriptional profiling. The quality of the RNA extracted from ARPE-19 cells treated with human vitreous reached the requirements for RNA sequencing (data not shown). Significant DEGs between the control and the vitreous-treated groups were identified based on the criteria of a log2-fold change of >2. The results showed that in the RPE cells treated with human vitreous there were 1,093 differential genes, of which there were 258 genes up-regulated (23.6%) and 835 genes were down-regulated (76.4%). Among them, there are three genes related to eye diseases (Figure 1). Cluster analysis was performed on 46 of the differentially expressed genes in the genomics data. In the cluster heatmap, the red and blue regions represent the up-regulated and down-regulated expression of genes, respectively (Figure 2); KEGG enrichment analysis revealed that nearly half of the differential genes were mainly concentrated in pathways related to cellular components such as the mitogen-activated protein kinase (MAPK) pathway (Figure 3).

### Vitreous Induces Changes in *IL13RA2* Expression of mRNA and Protein

**FIGURE 4:**
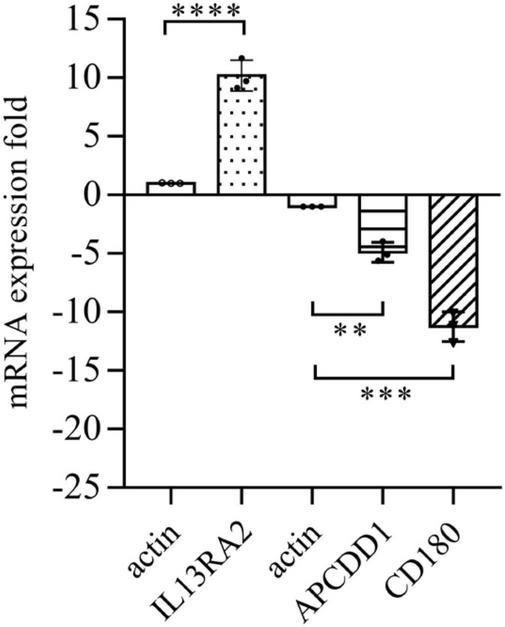
Vitreous induced changes in the mRNA expression. The qPCR analysis of mRNA expression in ARPE-19 cells induced by vitreous. The mean ± SD of three independent experiments is shown; ^****^ denotes 0.0001, using paired *t* test; ^***^ denotes 0.001, using paired *t* test; ^**^ denotes 0.01, using paired *t* test. Positive values indicate a fold increase in expression of the target gene relative to the internal reference, and negative values are opposite.

Among the 1,093 differential genes induced by vitreous, there were three genes with more than threefold changes (Table 1), which were confirmed by qPCR analysis (Figure 4). Noticeably, PVR is an inflammatory eye disease (12), IL13 is present in the vitreous (32); in addition, IL13Rα2 can bind to IL13 with high affinity to enhance cell proliferation and migration in the carcinogenesis (33, 34), and these cellular events are related to PVR pathogenesis. Thereby, we next investigated whether a vitreous-induced change in *IL13RA2* revealed by RNA sequencing was indeed the case. To this end, we treated ARPE-19 cells again with human vitreous and analyzed them with qPCR. The results showed that the vitreous treatments dramatically heightened the IL13RA2 expression in the RPE cells as shown in Figure 4.

**TABLE 1:** Main DEGs after vitreous induced.

Gene	Description	logFC	*P* value
CD180	CD180 molecule	−5.6276	0.0000
APCDD	APC down-regulated 1	−3.3411	0.0000
IL13RA2	interleukin 13 receptor subunit alpha 2	3.3898	0.0000

**FIGURE 5:**
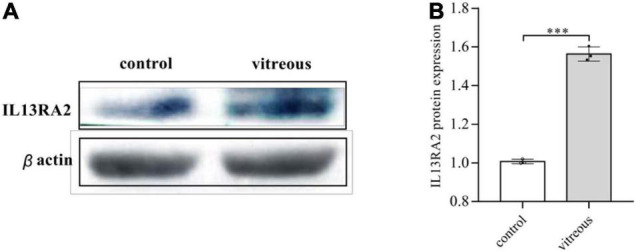
Vitreous induced the protein expression of IL13Rα2. Western blot analysis of the vitreous-induced protein expression of IL13RA2 in ARPE-19 cells. One of three representative experiments is shown. Bar graphs showed the Western blot band intensity, and the mean ± SD of three independent experiments is shown; ^***^ denotes 0.001, using paired *t* test. **(A)** The protein expression of IL13RA2 in ARPE-19 cells. **(B)** Histogram of protein expression gray value. The mean ± SD of three independent experiments is shown; ^***^ denotes 0.001, using paired *t* test. Control: ARPE-19 cells treated with DMEM/F12 only. Vitreous: ARPE-19 cells treated with vitreous diluted in DMEM/F12.

Since vitreous induced changes in mRNA expression of *IL13RA2*, we next examined if its protein levels were also changed with the vitreous stimulation. As expected, Western blot analysis showed that vitreous augmented IL13Rα2 expression in the ARPE-19 cells (Figure 5). This Western blotting data are consistent with those obtained from RNA sequencing and qPCR.

### IL13Rα2 Is Highly Expressed in Epiretinal Membranes From Patients With Proliferative Vitreoretinopathy

**FIGURE 6:**
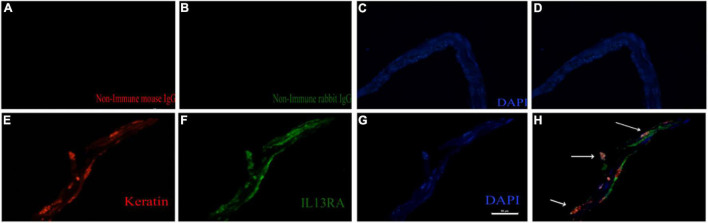
IL13Rα2 is highly expressed in RPE cells within epiretinal membranes from patients with proliferative vitreoretinopathy (PVR). Fibrotic epiretinal membranes from patients with PVR were first incubated with primary antibodies: a mixture of non-immune mouse and rabbit IgGs **(A–D)**, or of anti-IL13Rα2 and pan keratin antibodies **(E–H)** at 4°C overnight, and then with fluorescently labeled secondary antibodies at 1 h in room temperature. Co-staining of IL13Rα2 with pan keratin in H indicates IL13Rα2 expression in RPE cells in the ERMs from patients with PVR. Scale bar: 50 μm.

We next investigated whether IL13Rα2 was expressed in RPE cells within the epiretinal membranes from PVR patients. To this end, we stained epiretinal membranes with antibodies against keratin specific for epithelial cells and IL13Rα2. The immunofluorescence results showed that IL13Rα2 was highly expressed in RPE cells in epiretinal membranes from PVR patients (Figure 6).

### Neutralization of IL13Rα2 Prevents Vitreous-Induced Contraction of Retinal Pigment Epithelial Cells

**FIGURE 7:**
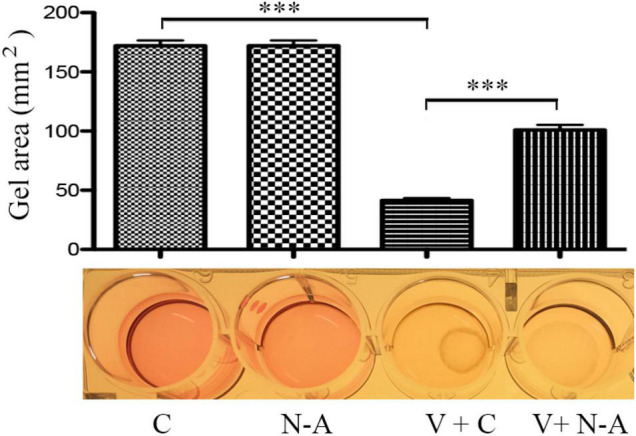
Blocking IL13Rα2 prevents vitreous-induced contraction of RPE cells. After the mixtures of ARPE-19 cells with collagen I solution formed a collagen gel, they were treated with DMEM/F12 + an antibody or vitreous + an antibody for 48 h. C, control antibody; N-A: neutralizing antibody against IL13Rα2; V + C, vitreous + control antibody; V + N-A, vitreous + neutralizing antibody against IL13Rα2. ^***^ denotes 0.001, using paired *t* test.

One of important events in the PVR pathogenesis is the contraction of epiretinal membranes, leading to the retinal detachment. Thereby we assessed if neutralization of IL13Rα2 could prevent vitreous-induced contraction of ARPE-19 cells in a collagen gel contraction assay, one of cellular models for PVR. As expected, vitreous stimulated the contraction of the collagen gel, and the antibody neutralizing IL13Rα2 significantly prevented the contraction induced by the vitreous (Figure 7), suggesting that this antibody is promising for PVR therapeutics.

## Discussion

In this article, we report that vitreous induced a large increase in IL13Rα2 in ARPE-19 cells and blocking IL13Rα2 with its neutralizing antibody prevents contraction of ARPE-19 cells, implicating a novel role of IL13Rα2 in the PVR pathogenesis. The clinical manifestations of PVR are related to a series of inflammatory and fibrotic changes (12, 35–37). When the retina of a patient ruptures, RPE cells exposed in the vitreous cavity respond to the vitreal growth factors and cytokines, leading to forward feedback to secrete these factors more (38), mediating wound repair responses such as matrix synthesis, cell migration, proliferation, and epithelial–mesenchymal transition (EMT), leading to the formation of epiretinal membranes, an essential process of PVR (39).

So far, the pathogenesis of PVR has not been completely understood. Hypotheses for PVR formation have been proposed for the essential roles of multiple growth factors and cytokines, such as transforming growth factor beta (TGF-β), platelet-derived growth factor (PDGF), vascular endothelial growth factors (VEGF), interleukins (ILs), tumor necrosis factor alpha (TNF-a) (12, 38, 40). ILs can be secreted by a variety of cells in response to a variety of stimulation including tissue damage. In recent years, studies have revealed that ILs are closely related to the occurrence and development of PVR. For instance, IL-6, a marker of acute inflammation (41), is up-regulated in the vitreous and subretinal fluid of the PVR group, and is positively correlated with the degree and duration of RRD and PVR grades (42, 43). IL-8, playing a key role in regulating inflammation and mediating angiogenesis, is elevated in PVR patients (44).

We herein report that IL13RA2 is heightened in the vitreous-treated RPE cells. IL13RA2, one of the high-affinity membrane receptors of IL-13, is highly expressed in tumors, such as liver cancer (45), glioblastoma (46), colon cancer (47), and pancreatic cancer (48). IL13RA2 has two forms: transmembrane form and extracellular soluble form (49–51). The transmembrane form is related to the signal transduction of ligands and the binding of other membrane receptors to form different functional subunits; soluble IL13RA2 seems to inhibit the function of IL13 (51, 52). It is speculated that the functions of the two forms of IL13RA2 may be antagonistic to each other. The previous studies on tumors reported that the overexpression of IL13RA2 endows tumors with the ability to invade and metastasize. IL-13 plays a key role in many pathological processes, such as asthma, pulmonary fibrosis, and ulcerative colitis (53, 54), and IL13 as an inflammatory factor plays an important part in various inflammatory reactions, suggesting IL13RA2 may play an important role in PVR.

**FIGURE 8:**
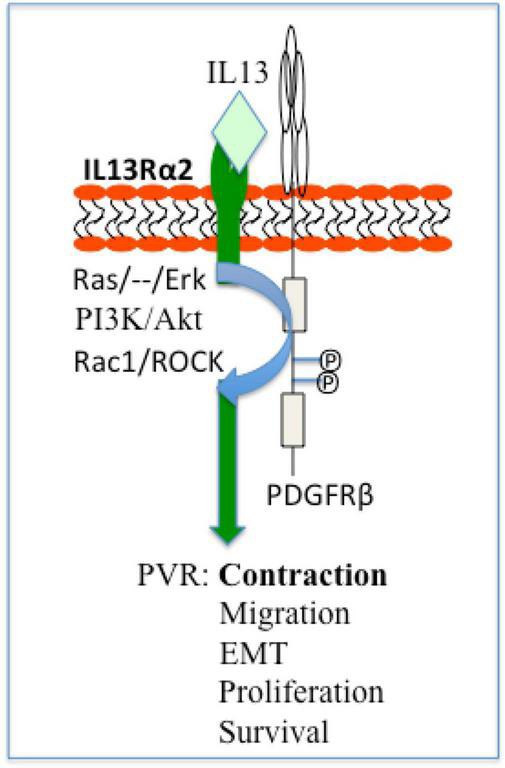
Schematic of a hypothesis: a role of IL13Rα2 cooperates with PDGFRβ in the PVR pathogenesis. Based on current literatures (13, 55) IL13Rα2 cooperates with PDGFRβ to initiate signaling pathways of Ras/Raf/MEK/ERK, PI3K/Akt, Rac1/ROCK, and thereby enhance cellular responses (survival, proliferation, EMT, migration, and contraction) intrinsic to PVR. ERK, extracellular signal-regulated kinases; PI3K, phosphoinositide 3 kinase; Rac1, Ras-related C3 botulinum toxin substrate 1; ROCK, Rho-associated protein kinase; EMT, epithelial–mesenchymal transition; PVR, proliferative vitreoretinopathy.

Noticeably, IL13Rα2 cooperates with epithermal growth factor receptor (EGFR) VIII signaling to promote glioblastoma multiforme (46), whereas PDGF receptor (PDGFR)β plays an essential role in vitreous-induced cellular responses related to PVR (13). Thereby we hypothesize that in ARPE-19 cells, IL13Rα2 interacts with PDGFRβ signaling to boost vitreous-stimulated cellular events intrinsic to PVR as illustrated in Figure 8, and this hypothesis is being tested in our research group.

In this study, we employed RNA sequencing to discover changes in vitreous-treated ARPE-19 cells, leading to our findings that IL13Rα2 was up-regulated significantly, and blockade of IL13Rα2 prevented contraction of ARPE-19 cells, suggesting that IL13Rα2 be a novel therapeutic target for PVR.

## Data Availability Statement

HL is the guarantor of this work, has full access to all the data in the study, and takes responsibility for the integrity of the data and the accuracy of the data analysis. The datasets generated during and/or analyzed during the current study are available from the corresponding author upon reasonable request.

## Ethics Statement

The studies involving human participants were reviewed and approved by the Jinan University Research Ethics Board. The patients/participants provided their written informed consent to participate in this study.

## Author Contributions

HQ, LD, and DF performed most of the experiments and analyzed the results. YW, NF, XM, GZ, and WW performed some experiments and managed projects. SZ and HL conceived the experiments, analyzed the data, and wrote the manuscript. All authors contributed to the article and approved the submitted version.

## Conflict of Interest

The authors declare that the research was conducted in the absence of any commercial or financial relationships that could be construed as a potential conflict of interest.

## Publisher’s Note

All claims expressed in this article are solely those of the authors and do not necessarily represent those of their affiliated organizations, or those of the publisher, the editors and the reviewers. Any product that may be evaluated in this article, or claim that may be made by its manufacturer, is not guaranteed or endorsed by the publisher.

## Abbreviations

ARPE-19: a human retinal pigment epithelial cell line
CLL: chronic lymphocytic leukemia
DEG: differentially expressed genes
EGFR: epidermal growth factor receptor
EMT: epithelial–mesenchymal transition
ERM: epiretinal membranes
HPRRE: human primary RPE
IL13RA2: Interleukin 13 receptor alpha-2
MAPK: mitogen-activated protein kinase
PDGFR: platelet-derived growth factor receptor
PVR: proliferative vitreoretinopathy
RPE: retinal pigment epithelial cells
TGF- β: transforming growth factor beta
TNF- α: tumor necrosis factor alpha
VEGF: vascular endothelial growth factor.
