# Supplementary material for: A Novel Role of IL13Rα2 in the Pathogenesis of Proliferative Vitreoretinopathy
Source: Front Med (Lausanne). 2022 Jun 13;9:831436. doi: 10.3389/fmed.2022.831436 (PMC9234175; doi:10.3389/fmed.2022.831436)
Supplement: Supplementary file 1 [file Data_Sheet_1.pdf]

Supplemental Data

Supplemental (s) Figure 1. Vitreous induced cell proliferation

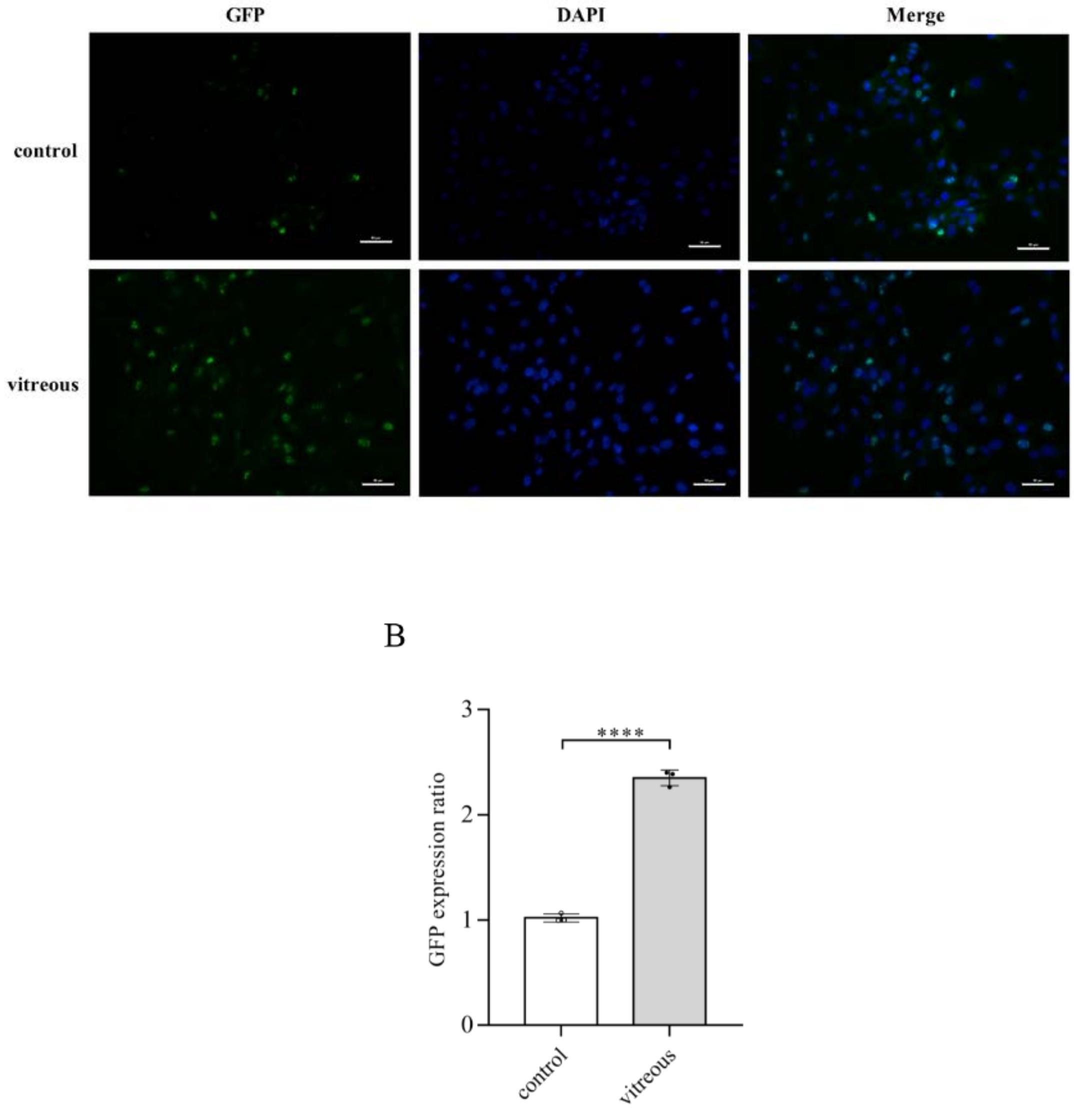

ARPE-19 cells in 24-well plates were treated with DMEM/F12 or vitreous diluted in DMEM/F12 (1:3). 24 hours later, the cells were stained with Ki67 for analyzing proliferation. (A): Ki67 staining result. Scale bar: 50μm. (B): Histogram of GFP expression ratio. The mean ± SD of three independent experiments is shown; **** denotes 0.0001, using paired t test. control: ARPE-19 cells. Vitreous: ARPE-19 treated with vitreous.

.

sFigure 2. Vitreous enhanced cell migration

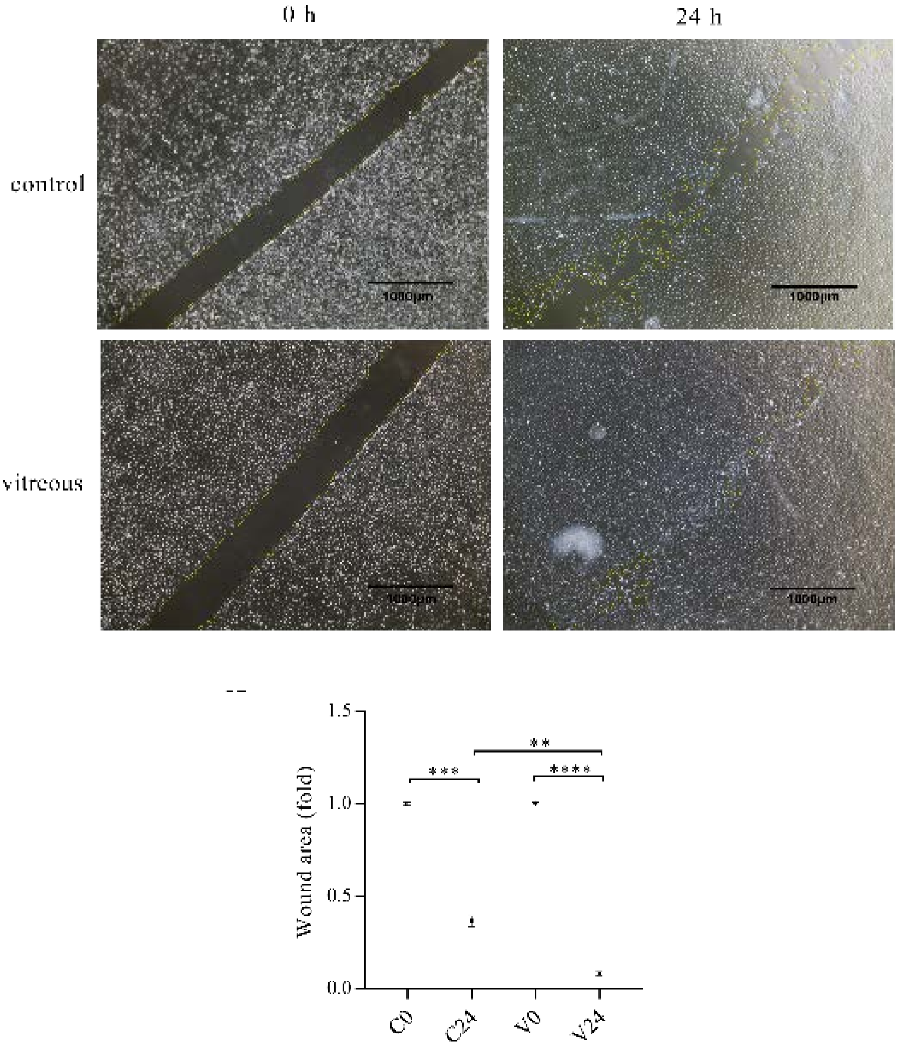

When ARPE-19 cells in a 12-well plate reached confluence, the cells were scratched with a 200 µl pipette tip and then treated with DMEM/F12 or vitreous diluted in DMEM/F12 (1:3). 24 hours later, the wounds were photographed, measured and analyzed using Adobe Photoshop 2018 software (top panel). The mean ± SD of three independent experiments is shown (Bottom panel). **, *** and **** denote p <0.01, p <0.001 and p <0.0001 using ordinary one-way analysis of variance. Representative raw data from three independent experiments are shown (below). Scale bar: 1000 µm.

sFigure 3. Vitreous promoted cell contraction

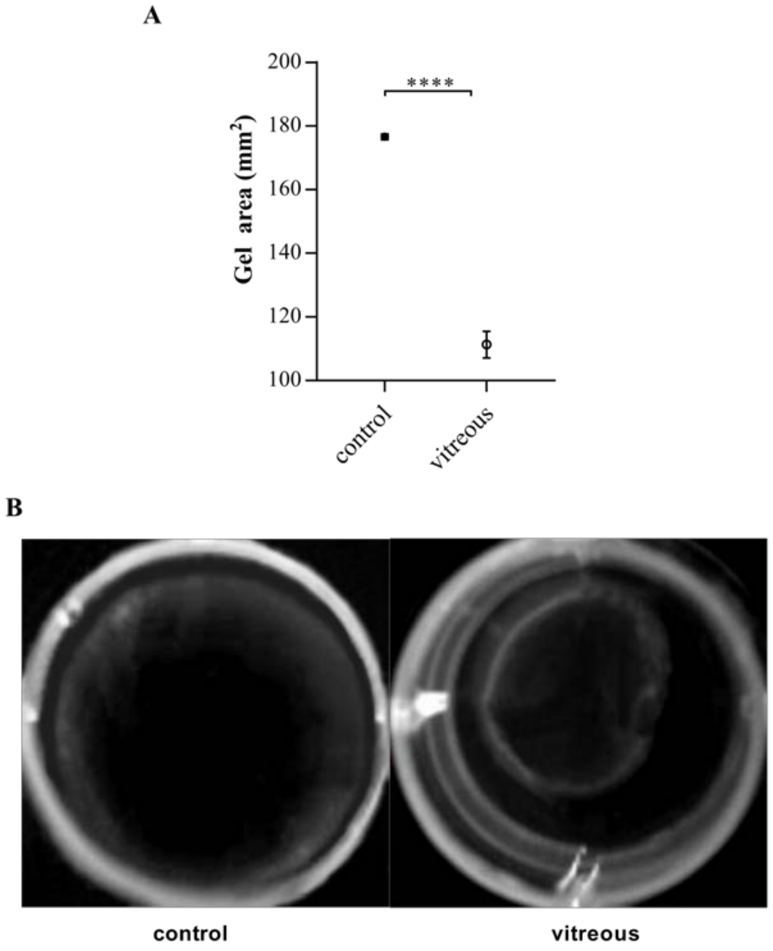

Mixture of ARPE-19 cells with collagen was transferred into a 24-well plate preincubated with BSA/PBS. After the gel was incubated at 37 °C for 90 minutes, DMEM/F12 alone (control), or DMEM/F12-vitreous was added onto the collagen gel. After 2 days, the gels were photographed for further analysis. The mean ± SD of the three in-dependent experiments is shown; *** denote $p < 0.001$, using a paired t test. A photograph of a representative experiment is shown at the bottom of the dot graphs.
